# The Role of Macrophages in Implementing the Effects of Secretome of Mesenchymal Stromal Cells in the Spermatogonial Stem Cell Niche

**DOI:** 10.17691/stm2025.17.2.04

**Published:** 2025-04-30

**Authors:** A.O. Monakova, N.A. Basalova, V.Yu. Balabanyan, K.L. Kryshen, A.A. Matichin, G.D. Sagaradze, V.S. Popov, A.Yu. Efimenko

**Affiliations:** PhD Student, Faculty of Medicine; Laboratory Assistant, Center for Regenerative Medicine; Medical Scientific and Educational Institute, Lomonosov Moscow State University, 27/10 Lomonosovsky Prospekt, Moscow, 119234, Russia; Junior Researcher, Center for Regenerative Medicine; Medical Scientific and Educational Institute, Lomonosov Moscow State University, 27/10 Lomonosovsky Prospekt, Moscow, 119234, Russia; Leading Researcher, Center for Regenerative Medicine; Medical Scientific and Educational Institute, Lomonosov Moscow State University, 27/10 Lomonosovsky Prospekt, Moscow, 119234, Russia; Head of Specific Toxicology and Microbiology Department; Research and Manufacturing Company “HOME OF PHARMACY”, 3/245 Zavodskaya St., Kuzmolovsky urban-type settlement, Vsevolozhsky District, Leningrad Region, 188663, Russia; Head of Specific Toxicology and Pharmacodynamics Department; Research and Manufacturing Company “HOME OF PHARMACY”, 3/245 Zavodskaya St., Kuzmolovsky urban-type settlement, Vsevolozhsky District, Leningrad Region, 188663, Russia; Junior Researcher, Center for Regenerative Medicine; Medical Scientific and Educational Institute, Lomonosov Moscow State University, 27/10 Lomonosovsky Prospekt, Moscow, 119234, Russia; Head of Interfaculty Scientific Research Laboratory of Translation Medicine, Faculty of Medicine; Leading Researcher, Center for Regenerative Medicine; Medical Scientific and Educational Institute, Lomonosov Moscow State University, 27/10 Lomonosovsky Prospekt, Moscow, 119234, Russia; Head of Laboratory of Tissue Repair and Regeneration, Center for Regenerative Medicine; Associate Professor, Biochemistry and Regenerative Biomedicine Department, Faculty of Medicine; Medical Scientific and Educational Institute, Lomonosov Moscow State University, 27/10 Lomonosovsky Prospekt, Moscow, 119234, Russia

**Keywords:** mesenchymal stromal cells, secretome, macrophages, spermatogonial stem cells, stem cell niche, spermatogenesis, immunotoxicity

## Abstract

Postnatal stem cells surrounded by a niche support the renewal and regeneration of tissues and organs throughout life. The dysfunction of niche components can lead to the development of diseases, which are hard to cure. We previously showed that the subtunical injection of mesenchymal stromal cell (MSC) secretome induced the spermatogonial stem cell niche restoration, although the mechanisms of the process are not fully revealed.

**The study aimed** at analyzing the effect of the MSC secretome on resident macrophages in animal models of male infertility and on peritoneal macrophages of intact animals.

**Materials and Methods:**

To study the effect of the MSC secretome on resident macrophages, doxorubicin-induced damage of murine spermatogenesis was modeled by intraperitoneal injections of 1 mg/kg of doxorubicin once in two days to reach a cumulative dose of 10 mg/kg. The second animal model of spermatogenesis injury was the abdominal cryptorchidism in rats. The MSC secretome was injected under the tunica albuginea. The animals were divided into the following groups: “intact”, “damage”, “MSC secretome”. After isolating the testes, the number of macrophages was estimated using the immunohistochemical analysis. To investigate the phagocytic activity of macrophages mice were intramuscularly injected into the thigh with the MSC secretome with the following isolation of peritoneal macrophages. The ability of peritoneal macrophages to absorb FITC latex particles was analyzed.

**Results:**

In cryptorchidism model the number of CD163^+^ M2 macrophages in the interstitium of testes increased significantly. The MSC secretome injection under the tunica albuginea of the testicle led to decreasing the number of CD163^+^ M2 macrophages. In the model of the toxic damage of spermatogenesis with doxorubicin the number of CD163^+^ M2 macrophages in the interstitium increased, however, there were no effects in the group with the MSC secretome injection. The number of M2 macrophages in this model, positive for another classical marker CD206, also increased, but the administration of MSC secretome reduced their number neither during the early nor in the late periods after damage. The study of MSC secretome effects on peritoneal macrophages demonstrated that a single intramuscular injection of MSC secretome in doses lower and higher than the therapeutic dose didn’t reduce, but conversely increased the phagocytic activity of macrophages.

**Conclusion:**

Our findings indicate the impact of the damage etiology and pathogenesis on the involvement of M2 macrophages in the implementation of the MSC secretome effects and the absence of its systemic immunotoxicity.

## Introduction

Acute or chronic injury of organs and tissues can result in severe diseases, and currently there are no effective treatment methods for many such disorders. In adults postnatal stem cells are primarily responsible for regeneration of injured structures. However, when their specific microenvironment (a niche of stem cells) is damaged, these cells die or decrease their ability to function. The niche recovery is a key stage for successful tissue regeneration. Active participants of the niche maintaining and regeneration are mesenchymal stromal cells (MSC), which are found in stem cell niches in almost all tissues in the human body. The regenerative mechanisms of MSC in the niche are various: from regulatory signals transmitted to the niche resident cells and recruiting functional cells to the niche up to direct differentiation into necessary cell types [1]. Frequently the regenerative effect of MSC is associated with secreting a complex of paracrine factors (secretome): growth factors, chemokines, cytokines, extracellular vesicles, extracellular matrix components [2]. The MSC secretome injected under the tunica albuginea in the animal model with damage to the spermatogonial stem cell (SSC) niche promoted the recovery of SSC niche functionality, and as a result, spermatogenesis recovery [3, 4]. The represented regenerative properties of the MSC secretome appear to be a promising basis for developing a biological medicinal product to treat male infertility of nonobstructive genesis.

The study of the MSC secretome mechanisms of action revealed its effect on the main resident cells of the niche: SSC, Sertoli cells, and Leydig cells. However, oftentimes the most significant MSC partners in various tissue-specific niches are macrophages [5]. MSC and macrophages interact through cell-cell contacts, transfer of organelles and secretion of soluble factors [6]. Macrophages are known to frequently play a key role in fulfilling regenerative MSC potential. Macrophage elimination in the models of damaged lungs, intestine, kidneys, skin significantly decreases a positive effect of the administered exogenic MSC secretome [7–9]. Thus, despite the proved direct effect of MSC on certain resident cells in niches, the effects on macrophages can also be significant.

Resident macrophages are found in many niches of stem cells. By their phenotype, these macrophages are similar to M2 type exhibiting anti-inflammatory properties. In injuries accompanied by aseptic inflammation, monocytes are attracted from the bloodstream and then differentiate into M2 macrophages [10]. An increased number of macrophages is the necessary stage for successful inflammation resolution. However, long maintenance of high number of M2 macrophages can result in chronic inflammation development and further pathological remodeling of tissues [11, 12].

The hypothesis of this study is that one of the regenerative mechanisms of the MSC secretome can be a decreasing the number of M2 macrophages in SSC niche. The study included two animal models of spermatogenesis damage: the toxic damage by doxorubicin in mice and bilateral abdominal cryptorchidism in rats. The advantage of the models is complex and reversible SSC niche damage that enables to assess regenerative effects of MSC secretome on different SSC niche components including macrophages [13].

Despite the fact that a local decrease of excessive immune activity in a target-organ is an important condition for successful regeneration, a decreased physiological level of the body immune system activity (immunotoxicity) can lead to undesirable side-effects. The present study also analyzed the MSC secretome effect on phagocytic activity of peritoneal macrophages in healthy mice that is necessary for estimating the safety of MSC secretome as a base for a biological medicinal product.

**The investigation aimed** at analyzing the effect of the MSC secretome on resident macrophages in animal models of male infertility and on peritoneal macrophages of intact animals.

## Materials and Methods

### Isolation of mesenchymal stromal cells from human adipose tissue

MSC of human adipose tissue was received from the biobank of the Institute for Regenerative Medicine, Lomonosov Moscow State University, collection ID: MSU_MSC_AD (https://human.depo.msu.ru). The cells were cultivated in the medium (Mesenchymal Stem Cell maintenance medium; AdvanceSTEM, USA) with 10% nutritional supplements (Stem Cell Growth Supplement; AdvanceSTEM, USA). The medium was changed every 3^rd^ or 4^th^ day. The cells were passaged up to 4–5^th^ passage, when achieving about 80% confluent.

### Manufacturing of human mesenchymal stromal cell secretome

Adipose-derived mesenchymal stromal cells, which achieved 80% confluence, were washed three times at 4–5^th^ passage using Hank’s solution (PanEco, Russia) followed by adding to the cells DMEM medium with low-concentration glucose (DMEM-LG; Thermo Fisher Scientific, USA), and cultivated within 7 days. The conditioned medium (secretome) was collected and centrifuged for 10 min at 300 g to remove cell debris.

### Concentration of mesenchymal stromal cell secretome

The concentration was performed on filters for the centrifuge Vivaspin turbo 15 (Sartorius, Great Britain) with a 10 kDa molecular weight cutoff. The filters were washed three times in 2% solution of penicillin– streptomycin in phosphate-buffered saline. The MSC secretome was concentrated 50-fold with filters by centrifugation at +4°С and at 3000 g. The obtained concentrated MSC secretome was used for further experiments.

### Animals

The animals were kept in standard conditions in accordance with Directive 2010/63/EU of the European Parliament and Council of the European Union dated September 22, 2010, about the protection of animals used for scientific purposes, as well as GOST 332152014 “Guidelines for accommodation and care of laboratory animals. Rules for equipping spaces and organizing procedures”.

#### Modeling of bilateral abdominal cryptorchidism

The study was carried out on adult male rats aged 3.5–4.0 months. For cryptorchidism modelling we used the previously proved technique [3]. The model is appropriate for developing a reversible SSC niche injury [14]. The testicles through the inguinal canal were drawn up from the scrotum to the abdominal cavity, then fixed by interrupted suture to the abdominal wall in the lateral canals area using atraumatic suture Prolene 4/0. The suture passed through the distal testicle pole to prevent possible blockage of the communication between the efferent ductules of testis and the seminiferous tubule. Special attention was paid to the avoidance of blood vessels and the deferent duct captured by suture. On day 15 of the experiment (2 weeks after the experiment started) the testes were brought down to the scrotum by the stay ligature removal followed by replacing the testes to the inguinal canal and administration of the drug based MSC secretome. Thus, the animals were divided into 3 groups: “intact” (no manipulation), “damage” (with cryptorchidism), “MSC secretome” (the animals with cryptorchidism were injected with MSC secretome at a dose of 10 U/animal). The MSC secretome (100 μl per testicle) was subtunically injected alongside with drawing down testes. Before administration, the MSC secretome was mixed with collagen gel “Applycoll” (purified porcine collagen type I; MakMedi, Russia) in a 1:4 ratio. After 1 month or 3 months the animals were euthanased using carbon dioxide (СО_2_) with the following death coming control. Such periods were chosen considering the spermatogenesis cycle duration, which is 54 days in rats. The chosen euthanasia times: 1 month (30 days) and 3 months (90 days) enable to assess the spermatogenesis before and after the whole cycle is completed [15]. After euthanasia the testes of male rats were isolated for further analysis.

#### Modeling of toxic spermatogenesis damage by doxorubicin

The investigation was carried out on adult male mice, C57Bl/6 line, aged 8–10 weeks. Doxorubicin was used as a damaging agent. The main mechanism of doxorubicin effect is DNA and RNA synthesis suppression: intercalation into duplex DNA between the pairs of nitrogenous bases following damage and changing of the structure of nucleic acids. Therefore, doxorubicin damages quickly dividing cells, including SSC resulting in male infertility [16]. Doxorubicin advantage for modeling compared to other chemotherapeutic agents is the effect on different cells of the niche providing the damage complexity and the ability to repel the pleiotropy of the MSC secretome effect. In addition, doxorubicin action is reversible [13].

The pilot studies revealed an optimal doxorubicin dose. The findings of histological analysis and analysis of spermatozoa showed the dose of 1 mg/kg sufficient for reversible damage. Doxorubicin hydrochloride was administered at a dose of 1 mg/kg once in two days. The total dose of the preparation administered was 10 mg/kg. Thus, the animals were divided into 3 groups: “intact” (no manipulations), “damage” (with toxic damage), “MSC secretome” (the animals with toxic damage were injected with the MSC secretome at a dose of 10 U/animal). The MSC secretome was administered after a doxorubicin damage under the tunica albuginea in the volume of 50 μl per testicle. Before administration, MSC secretome was mixed with collagen gel “Applycoll” in a 1:4 ratio. After 5 weeks or 5 months the animals were euthanased using carbon dioxide (СО_2_) with the following death coming control. Such periods were chosen considering the spermatogenesis cycle duration, which is 5 weeks (35 days) in mice. The chosen euthanasia terms correspond to 1 spermatogenesis cycle (35 days) and 4–5 spermatogenesis cycles (5 weeks). After euthanasia the testes of the male mice were isolated for further analysis.

#### The assessment of latex particle phagocytosis by murine peritoneal macrophages

The effects of the drug based on MSC secretome on the phagocytic activity of peritoneal macrophages were assessed in accordance with recommendations on estimating immunotoxic properties of medicinal products [17]. The investigation was carried out on adult C57Bl/6 male mice (STEZAR, Russia) aged 8–10 weeks and weighing 16–25 g. There were 3 experimental groups: “intact”, “intact + MSC secretome 250 U kg”, “intact + MSC secretome 416.5 U/kg”. The doses were in accordance with GOST 33215-2014 “Guidelines for accommodation and care of laboratory animals. Rules for equipping spaces and organizing procedures” were lower and higher than a therapeutic dose (10 U/animal), amounting 7.5 and 12.5 U/animal. For mice weighing 30 g, the MSC secretome doses for intramuscular administration were 250 U/kg (7.5 U/animal/30 g·1000 g) and 416.5 U/kg (12.5 U/animal/30 g·1000 g). Recalculation considering the animal weight in the experiment is needed due to systemic intramuscular administration of the MSC secretome. After MSC secretome administration the animals were given single intermittent (three portions, the time interval being 1 h) doses intraperitoneally; on day 8 of the experiment the animals were injected with 3 ml of 3% thioglycolic medium (State Scientific Center of Applied Microbiology and Biotechnology, Russia) in order to increase the number of released macrophages.

On day 11 of the experiment, the peritoneal exudate was drawn. For this purpose, the mice were euthanased using carbon dioxide (CO_2_) followed by cervical dislocation. The following procedures were performed in laminar air flow to achieve maximally sterile conditions. The abdominal wall was treated with 70% ethanol; then on the anterior abdominal wall the skin was cut. Saline solution (10 ml) of room temperature was injected into the abdominal cavity using a syringe. The abdominal cavity was massaged; then the exudate was drawn into 15-ml tubes. The procedure was repeated 5–6 times, the volume of the administered saline solution being 5 ml. The tubes with exudate were centrifuged using centrifuge СМ-6МТ (ELMI SIA, Latvia) for 10 min at 300 g.

### The study of phagocytic activity of macrophages

The sediment was resuspended in DMEM medium with L-glutamine (series DM-21-02 HS; Biolot, Russia) containing 10% FBS (series RE0000002; HyClone, USA), 10 ml. Using the hematological analyzer Mythic 18 Vet (Orphee, Switzerland), we determined the absolute number of macrophages. On average, in mice there were isolated about 30 million macrophages. For further procedures under aseptic conditions, we placed two cover glasses on the bottom of Petri dishes. The cell suspension from each animal (volume: 10 ml) was seeded into Petri dishes (1 Petri dish per 1 animal) and placed into the CO_2_-incubator at 37°С and 5% CO_2_ for 2 h. During incubation we prepared DMEM medium containing latex particles, 1 μm in diameter, conjugated with FITC (series MKCL6564; Sigma-Aldrich, USA) calculated as 100 latex particles per 1 macrophage. Non-adherent cells were drained. The remaining cells were washed four times using 10-ml saline solution, then added 10 ml of the medium with latex particles, and incubated for 3 h at 37°С and 5% СO_2_. After the incubation the Petri dishes were washed 4 times using saline solution, 10 ml. The cover glasses were stained using Romanowsky–Giemza method (for the sake of convenience, the staining was performed immediately in the dish) and dried at room temperature.

For fluorescence analysis, the Petri dishes were added 5 ml of trypan blue (0.8 mg/ml) and incubated for 1 min to eliminate the fluorescence of the particles appeared to be outside the cells. After inactivating fluorescence, the cover glasses were placed on the object slide, and the microscopic analysis was performed using fluorescent (absorption 495 nm, emission 517 nm) and light microscopy (microscope Axio Lab.A1; Carl Zeiss Microscopy GmbH, Germany).

We calculated the number of particles absorbed by 100 macrophages taking into consideration the number of particles for each analyzed macrophage. There was calculated the phagocytic activity index equal to the ratio of the total number of absorbed particles and the number of macrophages.

### Immunohistological methods

The testes from animals were fixed in the 10% formalin solution for 24 h, and then embedded in paraffin. After that the transverse sections 1 μm thick were prepared and dewaxed by successively displacing them into a more diluted ethanol solution. Then the sections were conditioned in 0.1 M citrate buffer for 20 min at 98°C. The tissue sections were stained with DAPI (Sigma, Germany) — DNA marker, CD206 (ab64693; Abcam, USA) and CD163 (ab182422; Abcam, USA) — markers of M2 macrophages in accordance with the manufactures’ protocols.

A microscopy was performed using Leica DM600Β microscope equipped with Leica DFC 420Х camera (Leica Microsystems, Germany). For calculations we obtained not less than 5 representative photos from one section. The images were processed and analyzed using the program software LasX (Leica Microsystems, Germany) and FiJi.

### Statistical analysis

According to the descriptive statistics, the data were verified on compliance with the normal distribution law using Shapiro–Wilk’s W test. In normal distribution we calculated the mean value (M) and standard error of mean (SEM). For data, which did not correspond to normal distribution, were calculated as median (Ме) and quartile range [Q1; Q3]. Intergroup comparison was performed using parametric or non-parametric methods when there were obtained the distribution type data. One-factor dispersion analysis (ANOVA) with the following intergroup comparison (post hoc) using Tukey test to assess the data with normal distribution. For the data, which failed to correspond to normal distribution, we performed paired comparison using Mann–Whitney test and Kruskall–Wallis criterion. The differences were considered significant if p<0.05. For statistical analysis we used program software Statistica 10.0 (StatSoft, USA).

## Results

*The MSC secretome has no effect on the number of CD163^+^ M2 macrophages in the interstitium in cryptorchidism model at early injury stages, 1 month after MSC secretome injection.*

**Figure 1.**
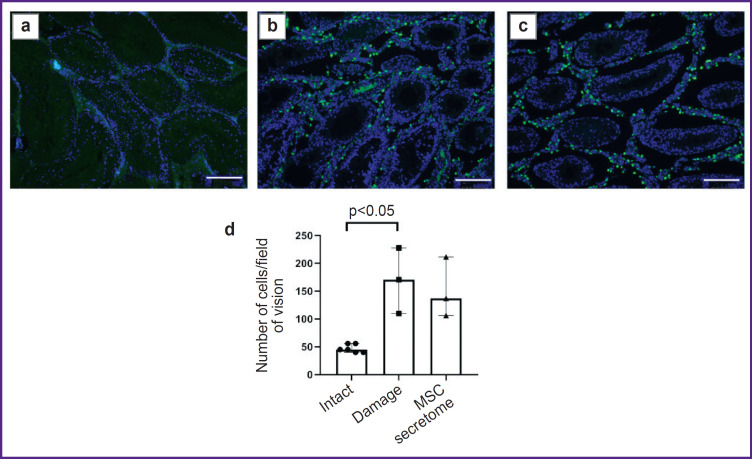
Tissue sections of male rats a month after the MSC secretome administration: (a)–(c) immunohistochemical staining by specific antibodies to CD163^+^; blue pseudo color — DAPI, nucleic acids marker; green pseudo color — CD163^+^ of M2 macrophages, primarily located in the interstitial space; (a) “intact” (no manipulations); (b) “damage” (with modelled cryptorchidism); (c) “MSC secretome” (the animals with cryptorchidism injected with MSC secretome); (d) the number of CD163^+^ M2 macrophages in field of vision; the number of testes under study in the “intact” group — 6, in the groups “damage” and “MSC secretome” — 3. The results are represented as median and 25^th^ and 75^th^ percentiles; for each section there were analyzed not less than 5 fields of vision. Bar — 200 μm

To study MSC secretome effects on the macrophages in testicles bilateral abdominal cryptorchidism in rats was performed. The testes elevated into the abdominal cavity for 2 weeks resulted in significant impairment of spermatogenic epithelium, interstitial hyperplasia, and significant increase in the number of CD163^+^ M2 macrophages in the interstitium. Previously, we showed the administration of MSC secretome under the tunica albuginea promoted the recovery of SSC niche structure and function [3]. At early stages there was no significant decrease in the number of CD163^+^ M2 macrophages in the testicles of animals with cryptorchidism and injection of MSC secretome (Figure 1).

*The MSC secretome contributes to the decrease in the number of CD163^+^ M2 macrophages in the interstitium in cryptorchidism model at late stages, 3 months after the MSC secretome injection.*

**Figure 2.**
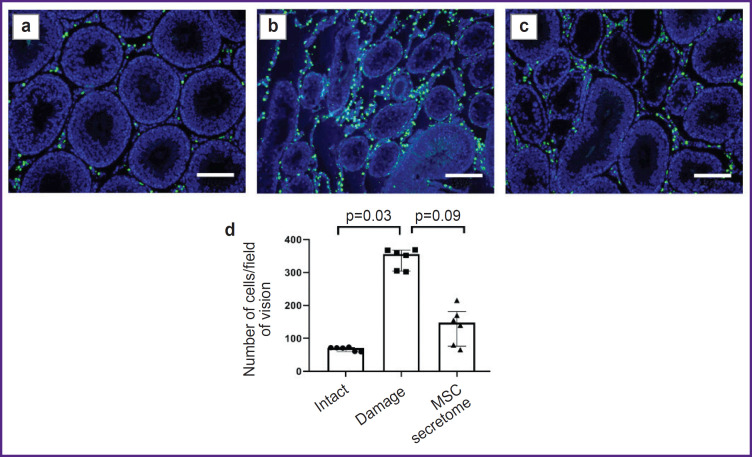
Tissue sections of male rats 3 months after the MSC secretome administration: (a)–(c) immunohistochemical staining by specific antibodies to CD163^+^; blue pseudo color — DAPI, nucleic acids marker; green pseudo color — CD163^+^ M2 macrophages, primarily located in the interstitial space; (a) “intact” (no manipulations); (b) “damage” (with modelled cryptorchidism); (c) “MSC secretome” (the animals with cryptorchidism injected with MSC secretome); (d) the number of CD163^+^ M2 macrophages in field of vision; the number of testes under study in each group — 6. The results are represented as median and 25^th^ and 75^th^ percentiles; for each section there were analyzed not less than 5 fields of vision. Bar — 200 μm

At late stage of cryptorchidism, 3 months after the MSC secretome administration, there was observed the decrease in the number of CD163^+^ M2 macrophages in the animal testicles. It can be an important mechanism of the MSC secretome action, mediating the niche recovery stimulation (Figure 2).

*The MSC secretome has no effect on the number of M2 macrophages in the interstitium in the doxorubicin- induced model of toxic injury of spermatogenesis at early injury stages, 5 weeks after the MSC secretome injection.*

**Figure 3.**
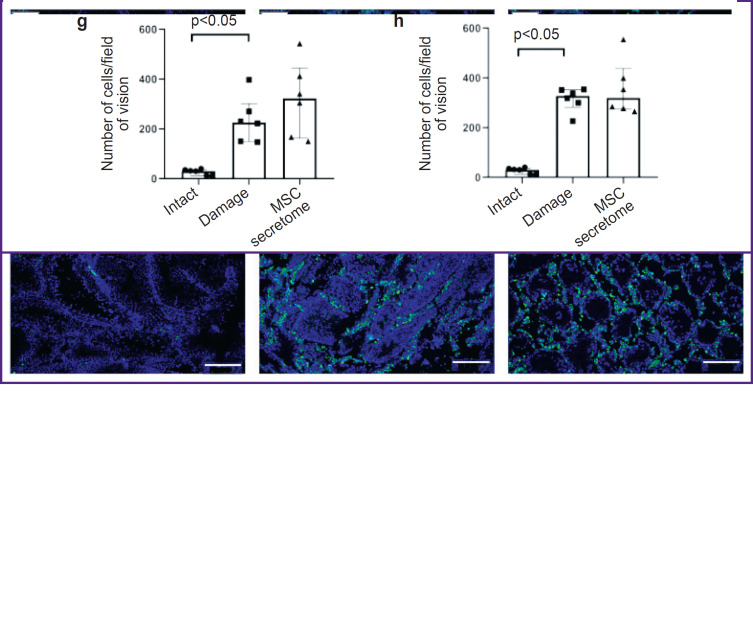
Tissue sections of male rats 5 weeks after MSC secretome administration: (a)–(f) immunohistochemical staining by specific antibodies to CD206^+^ and CD163^+^; blue pseudo color — DAPI, nucleic acids marker; green pseudo color — CD206^+^ and CD163^+^ M2 macrophages; (a)–(c) CD206^+^ M2 macrophages, primarily located in the interstitial space; (d)– (f) CD163^+^ M2 macrophages, primarily located in the interstitial space; (a) and (d) “intact” (no manipulations); (b) and (e) “damage” (with a toxic injury, without MSC secretome injection); (c) and (f) “MSC secretome” (the animals with a toxic damage injected with MSC secretome); (g) and (h) the number of CD163^+^ and CD206 M2 macrophages in field of vision, respectively; the number of testes under study in each group — 6; the results are represented as median and 25^th^ and 75^th^ percentiles; for each section there were analyzed not less than 5 fields of vision. Bar — 200 μm. *See the ending*

To prove the hypothesis on the MSC secretome effect on M2 macrophages in the damaged SSC niche, we modeled a toxic injury of spermatogenesis by doxorubicin in mice. After 5 weeks after doxorubicin injections, the number of CD163^+^ M2 macrophages in the testicular interstitium increased. However, the local administration of the MSC secretome had no effect on the number of macrophages (Figure 3 (g)). We also analyzed M2 macrophages in testicles using another classical marker of M2 macrophages — CD206, and the results were similar: after doxorubicin injection the number of CD206^+^ M2 macrophages significantly increased, while the MSC secretome administration had no effect on the number of CD206^+^ M2 macrophages (Figure 3 (h)).

*The MSC secretome has no effect on the number of M2 macrophages at late stages in the model of toxic injury by doxorubicin.*

**Figure 4.**
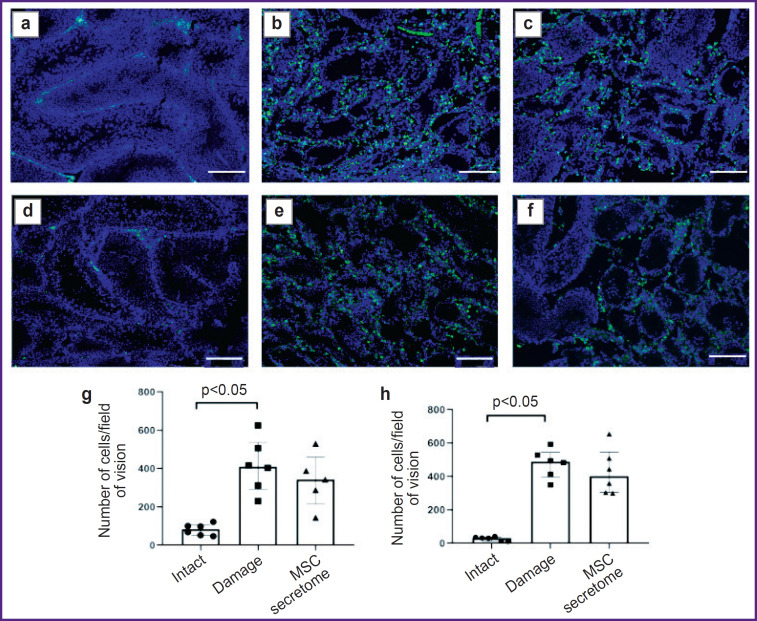
Tissue sections of male rats 5 months after MSC secretome administration: (a)–(f) immunohistochemical staining by specific antibodies to CD206^+^ and CD163^+^; blue pseudo color — DAPI, nucleic acids marker; green pseudo color — CD206^+^ and CD163^+^ M2 macrophages; (a)–(c) CD206^+^ M2 macrophages primarily located in the interstitial space; (d)– (f) CD163^+^ M2 macrophages, primarily located in the interstitial space; (a) and (d) “intact” (no manipulations); (b) and (e) “damage” (with a toxic injury, without MSC secretome injection); (c) and (f) “MSC secretome” (the animals with a toxic damage injected with MSC secretome); (g) and (h) the number of CD163^+^ and CD206 M2 macrophages in field of vision, respectively; the number of testes under study in each group — 6; the results are represented as median and 25^th^ and 75^th^ percentiles; for each section there were analyzed not less than 5 fields of vision. Bar — 200 μm

To make sure the MSC secretome injection under the tunica albuginea in the model of toxic spermatogenesis injury has no effect on M2 macrophages at later stages, we analyzed testicular sections 5 months after MSC secretome injection. The number of M2 macrophages in the damaged SSC niche significantly increased compared with the intact animals, however, the MSC secretome administration had no significant effect of their number (Figure 4).

*The MSC secretome exhibits no immunotoxicity towards macrophages.*

**Figure 5.**
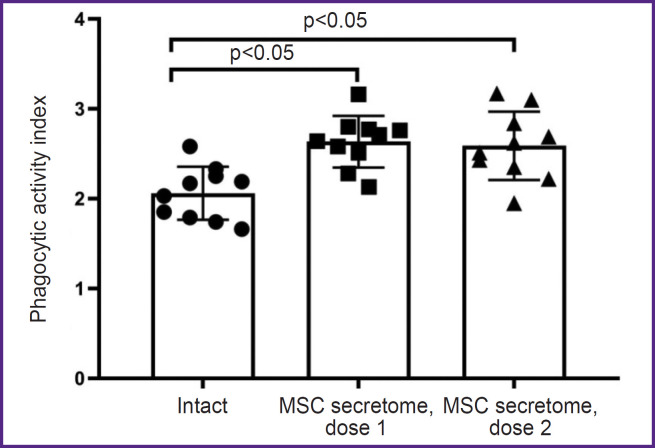
Phagocytic activity index of peritoneal macrophages The number of animals in each group — 10. The results are represented as mean and standard deviation; dose 1 — 250 U/kg (7.5 U/animal); dose 2 — 416.5 U/kg (12.5 U/animal)

The phagocytic activity of macrophages was studied by a classical test to assess a nonspecific component of the immune response using the absorption of latex particles conjugated with FITC. A single intramuscular injection of the MSC secretome at doses of 250 U/kg (7.5 U/animal) and 416.5 U/kg (12.5 U/animal) didn’t decrease the activity of systemic peritoneal macrophages, conversely, it appeared to increase their phagocytic activity (Figure 5).

## Discussion

Macrophages are important supporting cells in many niches of stem cells. The SSC niche was found to contain M2 macrophages as well, their leading role consisting in regulating other cells and providing SSC niche immune privilege [18]. Using two different models of SSC niche damage we showed that in case of damage the number of M2 macrophages positive by CD163 and CD206 markers in the interstitial space significantly increases. Our findings closely correspond to the data by other authors, who claim that the number of M2 macrophages in other spermatogenesis damage models also significantly increases compared to the intact animals [19–21]. Moreover, the number of CD163^+^ M2 macrophages was shown to decrease in cryptorchidism model rats at late stages of MSC secretome injection under the tunica albuginea that was associated with the recovery of the niche structure and reproductive function of male rats [3]. However, in the model of toxic damage of spermatogenesis in mice the number of CD163^+^ and CD206^+^ M2 macrophages had no significant changes both at early and late stages. The absence of effects on macrophages can be associated with differences in models and pathogeneses of the SSC niche damage. After doxorubicin administration the damage of seminiferous tubules appeared to be more pronounced than in cryptorchidism. Our previous study [4] on the model of toxic spermatogenesis damage demonstrated that the number of normal and recovering tubules did not significant differ from the group of animals without therapy despite the fact MSC secretome injection followed to significantly increasing the number of spermatozoa. In addition, on the cryptorchidism model after administration of MSC secretome the number of damaged tubules reduced, while the number of normal and recovering ones significantly increased [3]. Thus, the two models showed a different level of the SSC niche recovery, and therefore, the cell mechanisms of the niche recovery after MSC secretome injection are likely to be different as well that needs further studying.

Macrophages can be both: a direct target for the MSC secretome, and also the mediators in implementing its effects on other SSC niche cells. Some authors [22–24] showed an inhibiting action of macrophages *in vitro* on the secretory activity and cytoskeleton arrangement of Leydig cells. So, the decrease in the number of macrophages after the MSC secretome administration can promote the increase in the secretory activity of Leydig cells that is also important for normal functioning of the SSC niche.

The present study demonstrated that MSC secretome does not decrease the functional activity of peritoneal macrophages. Moreover, phagocytic activity of macrophages significantly increased in the group of animals injected with MSC secretome. Since MSC secretome contains the factors of human origin, increase of the phagocytic activity of peritoneal macrophages in mice can be associated with a natural immune reaction of the experimental animals in response to administration of foreign proteins.

## Conclusion

MSC secretome showed the local activity in relation to M2 macrophages on the rat cryptorchidism model. The local administration of MSC secretome to the damaged SSC niche resulted in a decrease in M2 macrophage number that could make a significant contribution to the niche recovery. However, the effect is likely to depend on the genesis of SSC niche damage. It is important to notice that the MSC secretome does not inhibit the functional activity of peritoneal macrophages, having no toxic effect on a macrophagal component of the immune system. The obtained data revealing the MSC secretome effect on M2 macrophages and absence of immunotoxicity have great importance for its further translation into clinical practice.
